# Effects of physical activity in child and adolescent depression and anxiety: role of inflammatory cytokines and stress-related peptide hormones

**DOI:** 10.3389/fnins.2023.1234409

**Published:** 2023-08-28

**Authors:** Shaojuan Hu, Xinyuan Li, Luodan Yang

**Affiliations:** ^1^College of Physical Education and Sports Science, Hengyang Normal University, Hengyang, China; ^2^College of Physical Education and Sport Science, South China Normal University, Guangzhou, China

**Keywords:** physical activity, adolescent, depression, anxiety, neurobiological markers, HPA axis

## Abstract

Depression and anxiety are the most common mental illnesses affecting children and adolescents, significantly harming their well-being. Research has shown that regular physical activity can promote cognitive, emotional, fundamental movement skills, and motor coordination, as a preventative measure for depression while reducing the suicide rate. However, little is known about the potential role of physical activity in adolescent depression and anxiety. The studies reviewed in this paper suggest that exercise can be an effective adjunctive treatment to improve depressive and anxiety symptoms in adolescents, although research on its neurobiological effects remains limited.

## 1. Introduction

Depression and anxiety are the most common mental health conditions affecting adolescents and is recognized as a disorder with a more significant burden worldwide than ischemic heart disease, cerebrovascular disease, and tuberculosis (Adam et al., 2010; Vrshek-Schallhorn et al., 2013). Stressful life events cause depression and anxiety in adolescents, including intimate social groups, family relationships, academic pressure, and health (Adam et al., 2010; Vrshek-Schallhorn et al., 2013). Stress contributes to increasing depression common among adolescents worldwide. Statistically, more than 75% of adults with fear- or anxiety-related disorders suffered from mental disorders during childhood or adolescence (Kim-Cohen et al., 2003). More than 10% of adolescents are diagnosed with depression and anxiety disorders in the United States (Costello et al., 2005; Kessler et al., 2005), and approximately 20% of adolescents with mental illnesses persist into adulthood (Paus et al., 2008). Depression and anxiety are common mental disorders and the risk factor for suicide. Investigating the non-invasive approaches to preventing and treating depression and anxiety is valuable for reducing the medical and social burden.

Physical activity has been shown to be effective in treating depression and anxiety in adults. In the general population, it has been shown to positively affect neurogenesis in crucial areas of the brain, including the hippocampus, and positively correlate with mental health in children, adults, and older adults. Additionally, exercise has many positive effects on brain health and cognitive function (Cotman et al., 2007; Mattson, 2012). The beneficial effects of physical activity on brain disorders have been widely studied (Aarsland et al., 2010; Williams et al., 2010; Pedersen and Saltin, 2015; Santos-Lozano et al., 2016). For example, physical activity reduces the risk of several neurodegenerative diseases (Aarsland et al., 2010; Williams et al., 2010; Pedersen and Saltin, 2015; Santos-Lozano et al., 2016). Exercise inhibits cognitive decline in people with neurodegenerative diseases and healthy individuals of all ages and has a lifelong positive effect on stress, anxiety, and depression (Voss et al., 1985; Pedersen and Saltin, 2015). However, there is relatively little data available on the impact of physical activity on adolescents and youths with mental disorders.

In addition, psychotherapy, psychosocial therapy (e.g., cognitive-behavioral therapy), and biological treatments (e.g., selective serotonin reuptake inhibitors [SSRIs] or tricyclic drugs) are the most common treatments. The use of safe and appropriate alternatives is essential in treating adolescent psychiatric disorders due to concerns about the adverse consequences of medication for depression in adolescents. Therefore, in this review, we summarized the effects of physical exercise on the child and adolescents’ depression and anxiety and discussed the role of inflammatory cytokines and stress-related peptide hormones.

A narrative review of the literature was conducted on physical activity and adolescent depression and anxiety. PubMed, MEDLINE, Web of Science, and Scopus databases were used. Literature with clear descriptions of physical activity, depression and anxiety, and adolescent were included. This review mainly focused on the role of inflammatory cytokines and stress-related peptide hormones in adolescent depression and anxiety.

## 2. Adolescent depression and anxiety

Most current studies examining risk factors for depression have involved adults (Lopez-Duran et al., 2009; Dowlati et al., 2010; Miller et al., 2015; D'Acunto et al., 2019; Nikkheslat et al., 2020). However, biomarkers of the risk of depression in adolescence and young adulthood have not been identified or validated (Lopez-Duran et al., 2009; Miller et al., 2015; D'Acunto et al., 2019). The etiology and pathophysiology of depression are complex and multifactorial. The transition from childhood to adulthood is characterized by many physical, behavioral, and neurological changes, especially the more remarkable plasticity and flexibility of neuropathy during adolescence (Lee and Gorzalka, 2015), increasing the risk of external interventions such as stress exposure or drug use in adolescents. A large body of evidence suggests that factors such as adolescent stress exposure have a more lasting and detrimental effect on the stress response than stress exposure in adulthood. Depression has a high incidence in childhood and adolescence and is chronic throughout life, making adolescence a crucial time to develop effective prevention strategies and to reduce the burden associated with depression in adulthood. Physical exercise is inexpensive and has almost no side effects (except for high-intensity exercise-induced injury). However, there are still controversies about its potential use in preventing and treating adolescent depression, such as intensity and duration of exercise. Moreover, previous relevant studies may be limited by their method of control group selection.

## 3. Neurobiological mechanisms of adolescent depression and anxiety

Currently, the risk factors for depression have been studied mainly in terms of biological or environmental mechanisms (Lopez-Duran et al., 2009; D'Acunto et al., 2019; Tang et al., 2020), and many theories have been proposed to explain the biological pathways of depression in adults. These include the monoamines theory, increased immune system activation, and abnormalities in the hypothalamic–pituitary–adrenal (HPA) axis (a stress-related pathway) (Dowlati et al., 2010; Koss and Gunnar, 2018; Zhang et al., 2018; Kennis et al., 2020). The neuroendocrine biomarkers include the HPA axis (Koss and Gunnar, 2018; Zhang et al., 2018; Kennis et al., 2020), inflammation (O’Brien et al., 2007), endogenous cannabinoids (Hill et al., 2008; Zajkowska et al., 2014), vitamins, polyunsaturated fatty acids (Grosso et al., 2014), hormones (di Luigi et al., 2006; Koss and Gunnar, 2018), neurotrophic factors (Duman and Li, 2012; Archer et al., 2014), telomere/gene length (Simon et al., 2015), vitamin D (Menon et al., 2020), neuroplasticity, gene expression (including mRNA quantification), and brain-related abnormalities. Additionally, neuroimaging has proposed other potential biomarkers (e.g., frontal limbic dysfunction, hyperactivity of limbic brain structures, and underactivity of the prefrontal cortex) (Radley and Sawchenko, 2011; Herman, 2013). However, there is less data on adolescent depression, with the existing data mainly from the aspects that follow. The following section will discuss neurobiological mechanisms involved in adolescent depression and anxiety.

### 3.1. Hypothalamic-pituitary-adrenal axis

Due to the complexity of stressors, the responses of the body also involve multiple stress systems (Hostinar and Gunnar, 2013). The HPA axis is one of the significant response systems and responds to many stressors (Koss and Gunnar, 2018). The brain maintains homeostasis by promoting the feedback inhibition of the HPA axis, which is indispensable in terminating stress responses. HPA axis dysregulation plays a crucial role in the pathogenesis of depression (Zhang et al., 2018). The classical view is that the HPA axis is activated by internal or external stimuli, disrupting its homeostasis. Excitatory projections are sent from the amygdala, prefrontal cortex, and hippocampus to the paraventricular nucleus (PVN) of the hypothalamus, leading to the synthesis and release of corticotropin-releasing factor (CRF), which acts at the pituitary level to induce the production of adrenocorticotropic hormone (ACTH). ACTH then signals the adrenal glands to release glucocorticoids (corticosterone in rodents and cortisol [CORT] in humans), initiating the negative feedback loop of the HPA axis. Adrenal glucocorticoids also play a role in reducing the activation of the HPA axis at all levels (Nabkasorn et al., 2006; Stranahan et al., 2008; Lee and Gorzalka, 2015; Koss and Gunnar, 2018).

The sensitivity of the HPA axis to external pressure increases due to the immature negative feedback of CORT to ACTH and CRH, leading to an increase in the baseline level of CORT (Wulsin et al., 2016). Hyperactivity of the HPA axis and high CORT levels may be associated with depressive symptoms (Wulsin et al., 2016). Many brain regions contain glucocorticoid receptors, with higher densities in the amygdala and hippocampus. Late adolescent exposure to chronic variable stress (CVS) in female rats induced insufficient CORT and ACTH secretion and increased depression-like behaviors (Wulsin et al., 2016).

It has been found that HPA axis dysregulation in patients with depression is associated with CORT levels, possibly through the following pathways: Firstly, glucocorticoid resistance, i.e., glucocorticoid receptor-mediated negative feedback, is one of the main theories proposed for HPA axis dysregulation and elevated CORT levels in patients with depression (Cattaneo et al., 2020; Nikkheslat et al., 2020). CORT is the primary hormone of the HPA axis, and a key glucocorticoid produced in response to stress, regulating neuronal survival and neurogenesis. CORT receptors are present throughout the body, forming the end of the human HPA axis. Glucocorticoid resistance broadly impacts developmental processes and physical and mental health systems (Koss and Gunnar, 2018) and is a physiological risk factor for depression in adults (Zorn et al., 2017; Kennis et al., 2020). Secondly, high levels of circulating CORT can lead to reduced neurogenesis, leading to the onset of depression (Anacker, 2014).

Many studies have shown that stress-induced functions of the HPA axis differ between prepubertal adolescent and adult rodents (Lee and Gorzalka, 2015). Doremus-Fitzwater et al. used an acute restraint stress model in developing and adult male and female rats displaying anxiety. They found that adolescents were more sensitive to repetitive stress than adults, exhibiting weight loss, increased baseline plasma CORT, and slower recovery, with females being slower than males and adolescents slower than adults (Doremus-Fitzwater et al., 2009). The response of the adolescent HPA axis to stressors is prolonged (McCormick and Mathews, 2007), exhibiting significantly prolonged hormonal stress responses (e.g., ACTH, total corticosterone, and free corticosterone) (Romeo et al., 2006). However, it returns to the baseline more quickly (Gomez et al., 2002). These may alter behavioral responses to drugs and cognitive performance in adulthood (McCormick and Mathews, 2007). The prolonged release of corticosterone is due to the incompleteness of the HPA axis negative feedback rather than differences in adolescent corticosterone clearance rates or decreased adrenal sensitivity to ACTH (Gomez et al., 2002). These differences in puberty may be related to differential neuronal activation in the hypothalamic PVN (Romeo et al., 2006), indicating that experience-dependent neuroplasticity in the HPA axis neuroendocrine system is significantly correlated with pubertal maturation.

Furthermore, increased CRH neuronal activation in the prepubertal PVN was associated with neuronal activation in the corticolimbic circuitry but was not found in adult rats (Romeo et al., 2006). Rats exposed to CVS in late adolescence exhibited elevated basal corticosterone and oxytocin levels. Still, only the adult group for CVS showed somatic, HPA axis, and neuropeptide effects in the forced swimming test (Jankord et al., 2011).

CORT levels indicate HPA axis function, and both basal and diurnal CORT levels in adolescents differ from those in adults (Van Cauter et al., 1996; Guerry and Hastings, 2011). A meta-analysis by Zajkowska et al. showed that in adolescent populations with major depressive disorder (MDD), morning CORT levels were strongly associated with subsequent MDD development. Afternoon CORT levels and CORT stress responses did not differ between adolescents with MDD and healthy controls, and elevated morning CORT levels preceded subsequent MDD episodes. Thus, elevated CORT may predict depression (Zajkowska et al., 2022), and elevated morning CORT levels are associated with an increased risk of adolescent depression.

### 3.2. Inflammatory pathways

Smith originally proposed the “macrophage theory of depression,” suggesting that the excessive secretion of macrophage cytokines such as interleukin (IL)-1, tumor necrosis factor (TNF)-α, and interferon-c is associated with severe depression (O’Brien et al., 2007). Acute and chronic increases in pro-inflammatory cytokines can lead to the onset of a series of diseases with symptoms that overlap with those of major depression, including anhedonia, reduced activity, and social withdrawal (Dantzer et al., 2008; Shakhar and Shakhar, 2015). Some inflammatory markers interact with early life stress and can predict depression, such as TNF-α and IL-1β (Dantzer et al., 2008; Paolucci et al., 2018). When these cytokines are injected peripherally into the bloodstream, they enter the brain to activate microglia and amplify cytokine activity (Frank et al., 2007), which in turn leads to alterations in neurotransmitter metabolism, neuroendocrine function, and behavioral symptoms that are common in patients with severe depression, suggesting that they may contribute to the etiology of the disorder (Dantzer et al., 2008; Paolucci et al., 2018).

Another pro-inflammatory cytokine associated with depressive symptoms is IL-6. Patients with SSRI-resistant depression have significantly higher production of the peripheral pro-inflammatory cytokines IL-6 and TNF-α compared to normal controls (O’Brien et al., 2007). Hepatocytes produce C-reactive protein (CRP) in response to increases in IL-6, TNF-α, and IL-1β (McDade et al., 2006). Childhood traumatic events are recognized risk factors for depression and other mental illnesses and are associated with elevated levels of inflammatory markers such as CRP or IL-6 (Baumeister et al., 2016). Danese et al. also found significantly higher CRP levels in adolescents with depression who had experienced childhood physical abuse compared to healthy controls (Danese et al., 2011), suggesting that adolescent depression may be related to the immune system.

Bendezú et al. (2022) found that changes in peripheral IL-1β, IL-6, and TNF-α levels were positively correlated with depression and that the inflammatory stress response was concurrent with the HPA stress response after assays of peripheral pro-inflammatory cytokines (TNF-α, IL-1β, and IL-6) in adolescent girls with psychopathology risks. These changes may be due to early adversity leading to excessive activation of the sympathetic nervous system and the metastasis of innate immune cells, which causes an increase in peripheral inflammatory markers (Mondelli and Vernon, 2019). In contrast, activation of the innate immune system leads to structural abnormalities of the brain, such as a decrease in the hippocampal volume and a reduction in functional connectivity between the brain networks (Marsland et al., 2008; Mondelli and Vernon, 2019; Rasmussen et al., 2019; Kim et al., 2020).

In addition, excessive activation of the HPA axis can cause an increase in circulating inflammatory cytokines (Zajkowska et al., 2022), such as indoleamine-2-3-dioxygenase, an enzyme that causes a decrease in serotonin via the kynurenine pathway (Miller and Raison, 2016), and brain-derived neurotrophic factor (BDNF), which is thought to be involved in neuroprotection in depression and anxiety (Lee and Kim, 2010). Activation of the immune system may lead to brain-related abnormalities, including structural and functional changes in patients with depression (Opel et al., 2019).

### 3.3. Other biomarkers in peripheral blood and cerebrospinal fluid

The relationship between mood disorders and the endocrine system has been extensively studied in adults. The HPA, hypothalamic-pituitary-thyroid (HPT), hypothalamic-pituitary-gonadal axes, and hypothalamic growth hormone (GH) have been the research focus on adult depression. In examining whether severe depression in children and adolescents is the same as depression in adults, several peripheral blood and cerebrospinal fluid biomarkers were studied. For example, the 5-hydroxytryptamine and noradrenergic systems, which are associated with mood disorders, evolve significantly during childhood and adolescence and may play different roles in childhood and adult-onset mood disorders. However, searching for a biomarker that can replace cerebrospinal fluid is necessary due to the significant trauma and complications associated with its collection, which patients do not tolerate. Since peripheral blood and cerebrospinal fluid are in circulatory equilibrium, more studies focus on relevant peripheral blood biomarkers for a safer and quicker determination of mental disorders.

#### 3.3.1. Dexamethasone suppression test

Dexamethasone is a synthetic long-acting glucocorticoid that inhibits the production of CRH by the hypothalamus and ACTH by the pituitary gland. Although there is some controversy about the specificity of dexamethasone suppression tests (DSTs) in children and adolescents (Guerry and Hastings, 2011), in healthy adults, dexamethasone can inhibit the release of CORT. Dexamethasone non-suppression or early escape from dexamethasone suppression is thought to be partly the result of glucocorticoid receptor downregulation at hippocampal, hypothalamic, or pituitary levels and contributes to HPA axis hyperactivity in depressed adults (Zalsman et al., 2006; Guerry and Hastings, 2011; Wyller et al., 2016). In a test of dexamethasone suppression in children and adolescents, Lopez-Duran et al. found that children and adolescents with depression exhibited higher CORT production (or less suppression) after a DST compared to controls without depression and that, on average, approximately 45% of children and adolescents with depression and only 18% of control participants without depression had positive DST results in the mean of all studies (Lopez-Duran et al., 2009). These findings suggest that, unlike adults, the HPA axis in young adults with depression becomes active due to insensitivity to the hormonal “braking” system or early escape.

#### 3.3.2. Serotonergic system

Emerging studies found that the serotonergic system are associated with suicide, impulsive violence, and anxiety (Tyano et al., 2006). The HPT axis has a regulatory role in the 5-hydroxytryptamine system of the brain. Zalsman et al. measured plasma serotonin (p5-HT) to assess the relationship between p5-HT levels and psychometric measures. In the adolescent population, p5-HT levels were negatively correlated with suicide and violence in a subgroup of participants who were suicidal; however, p5-HT was higher in the entire clinical group compared with normal controls (Tyano et al., 2006). Therefore, the underlying mechanism needs to be further investigated.

Drugs that stimulate the serotonergic system result in the release of prolactin and CORT. In adult studies, the most commonly used drug was fenfluramine, which showed a weakened prolactin response in patients with depression compared to controls (Zhang et al., 2012). L-5-hydroxytryptamine (L-5-HTP) is a precursor of serotonin and increases serotonin turnover in the central nervous system. In a study of children with depression using L-5-HTP, Ryan et al. found reduced CORT levels but a higher prolactin response than normal controls and limited to girls with depression (Ryan et al., 1992). In comparing abused children with depression, unabused children with depression, and unabused normal controls, it was found that the abused and depressed group had increased prolactin release after L-5-HTP stimulation compared to the other two groups (Kaufman et al., 1998). These results suggest that aggressive and suicidal behavior in adolescents differs from that in adults. It is associated with more sensitive responsiveness of adolescent 5-hydroxytryptaminergic with 5-HT1A or 5-HT2 receptors, affecting their prolactin release. In addition, people with depression often suffer from disrupted sleep. However, no abnormal sleep was found in children and adolescents with depression. Emslie et al. (1990) found that hospitalized children with depression had shorter rapid eye movement (REM) latencies, longer sleep latencies, and more REM sleep time compared to normal controls, but no differences in stage 4 sleep, possibly related to less 5-HT neurotransmission or more central cholinergic activity (Zalsman et al., 2006).

#### 3.3.3. Growth hormone

Growth hormone (GH) is secreted by the anterior pituitary gland. Its secretion is stimulated by GH-releasing hormone and inhibited by somatostatin. Notably, Children with GH deficiency (GHD) exhibit psychological immaturity, which is manifested by a lack of social confidence and symptoms of depression (Akaltun et al., 2018). Akaltun studied 122 children and adolescents aged 7–17 years with GHD (of which 87 were treated for GHD, and 35 were untreated) and 122 healthy volunteers and found that children with GHD had significantly more severe generalized anxiety disorder and social anxiety disorder compared to healthy controls (Akaltun et al., 2018).

GH insensitivity may be a characteristic marker of depression. A distinctive feature of post-traumatic stress disorder (PTSD) is the hyperactivation of the sympathetic and noradrenergic systems. GH response to the α2 agonist clonidine can be assayed for noradrenergic receptor function. A study by Morris et al. (2004) to examine whether young subjects with combat-related PTSD (with or without comorbid depression) had an attenuated GH response to clonidine found that participants with depression and PTSD had slow GH responses to clonidine. This finding suggests that postsynaptic α2 receptors are sub-sensitive (Morris et al., 2004). A study by Fu et al. (2001) in which positron emission tomography scans were performed during clonidine stimulation found diminished noradrenergic function in the prefrontal cortex in depression, suggesting that GH sensitivity in depression is related to HPA function. Dahl et al. (1992) found no difference in GH levels between adolescents with MDD and the control. However, the MDD group with suicidal behavior (defined as having detailed plans or attempts) had reduced sleep GH secretion compared to the group without suicidal behavior. Abnormalities in the GH axis may be limited to preadolescent children with depression (Zalsman et al., 2006), but the role of GH in adolescent suicide is unclear and requires further study.

#### 3.3.4. Sex hormones

Pubertal maturation is a critical process in the development of the HPA axis, which can be influenced by sex hormone activation and their tissue effects. For example, the production and secretion of adrenal steroids in the HPA axis increase during early puberty, which stimulates the release of luteinizing hormone and follicle-stimulating hormone from the pituitary gland, thus promoting the production of gonadal steroids. The dramatic and rapid rise in gonadal hormones promotes reproductive maturation, physical growth, and the development of secondary sexual characteristics in adolescents. Elevated gonadal hormone levels also contribute to the organization and activation effects of the developing adolescent brain, which affects the HPA axis and may lead to the emergence of gender differences in HPA axis function. There is a great need to study the role of sex steroids and their potential mechanisms in developing the HPA axis.

di Luigi et al. (2006) examined the relationship between age 13.3 ± 2.1-year-old male adolescents before and after acute exercise to investigate the correlation between pubertal characteristics and salivary CORT (C), dehydroepiandrosterone sulfate (DHEAS), and DHEAS/C ratio. They found that after exercise-related stress, the mean pre-stress C and DHEAS concentrations significantly increased, while the DHEAS/C ratio significantly decreased. Pre-stress C was positively correlated with age, and changes in post-stress C concentration were negatively correlated with puberty, mean testicular volume, and pre-stress salivary DHEAS. In addition, salivary DHEAS concentrations and DHEAS/C ratios before and after exercise stress were positively associated with age, puberty, pre-stress salivary testosterone (T), testicular volume, and body mass index (di Luigi et al., 2006), suggesting that stress-related risk may be increased in males during early adolescence. It follows that differences in age at pubertal onset may also lead to different developmental trajectories of the HPA axis and ultimately contribute to gender differences in the HPA stress response.

In addition, there are other factors, such as endogenous cannabinoids (Hill et al., 2008; Zajkowska et al., 2014) and telomere length (Zajkowska et al., 2021), that are associated with mental disorders, all of which are somewhat controversial and lack clear clinical data support.

### 3.4. Effect of physical activity on adolescent depression and anxiety

#### 3.4.1. Effectiveness of physical activity on adolescent depression and anxiety

Numerous meta-analyses have shown that regular physical activity can improve various physiological and psychological factors in patients with depression (Kvam et al., 2016; Schuch et al., 2018). Exercise therapy has been shown to be effective in the treatment of depression in adults, and Kvam et al. included 23 randomized controlled trials and 977 participants in a meta-analysis, demonstrating that physical exercise is an effective intervention for the treatment of depression and may also be a viable adjunctive therapy in combination with anti-depressants (Kvam et al., 2016). In a meta-analysis of 49 prospective cohort studies (comprising 1,837,794 persons), Schuch et al. found that physical activity prevented the onset of depression regardless of age and geographic region (Schuch et al., 2018). However, fewer studies have been conducted on adolescent inpatients with depression. Philippot et al. assessed depression and anxiety symptoms using the Hospital Anxiety Depression Scale in 52 adolescent inpatients who were physically active three to four times a week for 6 weeks (a total of 20 h), concluding that planned physical exercise can be used as a complementary measure to adolescent psychiatric inpatient treatment to reduce their depressive symptoms (Philippot et al., 2022) and demonstrating the effectiveness of physical exercise in adolescent depression inpatient treatment. Another meta-analysis of 115,540 children and adolescents from 12 countries found a good correlation between meeting all three exercise recommendations in the 24-h guidelines and better mental health indicators in children and adolescents (Sampasa-Kanyinga et al., 2020). Several meta-analyses of exercise interventions for adolescents with depression have similarly concluded that physical activity is a promising primary intervention for adolescents with a diagnosis or threshold of symptoms of depression (Carter et al., 2016; Bailey et al., 2018).

However, the results from different studies varied. In a meta-analysis that included 16 studies with a total of 1,191 participants aged 11–19 years, Larun et al. showed that exercise had a small effect in reducing depression and anxiety scores compared to the general population of children and adolescents, regardless of whether the intensity of exercise was high or low (Larun et al., 2006). Also, in another meta-analysis that included 13,307 references with participants aged 12–18 years and predominantly female, it was found that although exercise interventions may be associated with a reduction in the severity of depression in adolescents, data were scarce. One of the trials found no significant decrease in the severity of depression at a six-month follow-up (Axelsdóttir et al., 2021). The limited number of studies and the clinical diversity of participants, interventions, and measurement methods may limit the drawing of conclusions. Therefore, the effect of exercise on treating anxiety and depression in children and adolescents remains unclear due to the lack of evidence. Because of the limited assessment of the effects of exercise therapy, the actual impact can vary greatly. Therefore, large-scale, high-quality trials of physical activity interventions for adolescent mental disorders, including follow-up periods, are required.

#### 3.4.2. Effect of physical activity intensity on adolescent depression and anxiety

Paolucci et al. (2018) studied 61 college students who underwent 6 weeks of high-intensity interval training (HIT), moderate continuous training (MCT), or no exercise (CON) during the semester. They found increased depression in the CON group and decreased depression in both the MCT and HIT groups. However, MCT reduced the levels of the pro-inflammatory cytokine TNF-α, while HIT increased perceived stress, TNF-α, and IL-6 (Paolucci et al., 2018). These changes may be due to higher levels of physical stress caused by a strenuous physical exercise. Imboden et al. conducted an experiment where patients with depression between 18 and 60 years of age were assigned aerobic exercise (AE) and basic stretching activities (control group) three times a week for 6 weeks. The severity of depression was assessed by the Hamilton Depression Rating Scale and Beck Depression Inventory, and the results showed that AE significantly reduced depression and had additional benefits on memory function compared to stretching exercises. Another meta-analysis that included eight randomized controlled trials also demonstrated that moderate-to-high intensity aerobic exercise is effective in reducing symptoms such as attention deficiency, hyperactivity, impulsivity, anxiety, executive function, and social barriers in children with attention-deficit/hyperactivity disorder (Cerrillo-Urbina et al., 2015).

#### 3.4.3. Mechanism of physical activity’s effects on adolescent depression and anxiety

Regular physical activity has been repeatedly shown to promote cognitive, emotional, and motor benefits while reducing pain and adverse effects. It is preventive in anxiety and depressive states and supports mental health in adolescents and adults. Exercise improves several biomarkers associated with depressive states: HPA axis homeostasis, anti-neurodegenerative effects, monoamine metabolic regulation, and neuroimmune function. Physical activity can serve as a “scaffold” to support the anti-inflammatory defense and nerve repair processes of BDNF (Archer et al., 2014).

Physical activity has different effects on the various levels of the HPA axis. It was found that wheel running can reduce the binding and expression of the mineralocorticoid receptors in mice’s hippocampus and increase the glucocorticoid receptors’ levels in rats. Running exercise can reduce CRF mRNA in the PVN, thereby limiting the increase of proopiomelanocortin mRNA in the anterior pituitary, activating tyrosine hydroxylase mRNA in the adrenal glands, and increasing corticosterone levels (Stranahan et al., 2008). Eight weeks of moderate-intensity group jogging significantly reduces 24-h urinary CORT and adrenaline secretion in young women with moderate depression (Nabkasorn et al., 2006). Aerobic running training for 120 consecutive days maintained the stability of BDNF, insulin-like growth factor 1, vascular endothelial growth factor, and C, preventing physical and psychological deterioration (Abeln et al., 2022).

The amygdala and hippocampus are critical centers in the limbic system. Both structures are connected to the HPA axis and can regulate the HPA stress response. The amygdala is primarily excitatory for HPA axis function, while the hippocampus is predominantly inhibitory (Radley and Sawchenko, 2011; Herman, 2013). Furukawa et al. (2021) found that exercise promotes increased adult hippocampal neurogenesis by releasing the systemic antioxidant selenium transporter protein selenoprotein P, which activates the quiescent hippocampal neural precursor cells. This finding suggests that physical exercise may promote increased hippocampal neurogenesis in the brain and play a specific role as an “exercise pill” in fighting depression. The medial prefrontal cortex (mPFC) responds to traumatic events by modulating the amygdala’s response, and different mPFC regions are associated with different roles in regulating the HPA axis (Murphy et al., 2022). Yan et al. (2022) found that exercise mediated the restoration of m6A in the mPFC and promoted mPFC activity to achieve anti-anxiety effects in mice.

The levels of other anti-depressant hormones related to physical activities, including salivary and plasma steroid hormones (CORT, T, and DHEA) and GH, correlate with the type, duration, and intensity of exercise. A systematic review found preliminary evidence that one-off acute exercise may exert its anti-depressant effects by increasing cardiac natriuretic peptide, brain natriuretic peptide, copeptin, and GH in patients with severe depression (Schuch et al., 2016). In contrast, long-term exercise may exert its anti-depressant effects by promoting long-term adaptation of copeptin, thiobarbituric acid reactive species, and total mean frequency. A recent acute exercise study (Kallies et al., 2019) and long-term randomized controlled trial (Kerling et al., 2017) suggest that aerobic exercise may exert its effects by increasing BDNF. However, a preliminary meta-analysis on only six randomized controlled trials and 176 participants (Dinoff et al., 2018) found no significant long-term change in BDNF.

Paolucci et al. (2018) studied the relief of depression through exercises from an inflammatory factor perspective in 61 college students who underwent 6 weeks of HIT, MCT, or no exercise (CON) during the semester. They found increased depression in the CON group but decreased depression in both the MCT and HIT groups and decreased levels of the pro-inflammatory cytokine TNF-α in the MCT group. However, physical activity also increased perceived stress, TNF-α, and IL-6 relative to MCT (Paolucci et al., 2018). These changes may be due to higher levels of physical stress caused by a more strenuous exercise regimen.

## 4. Discussion

**Figure 1:**
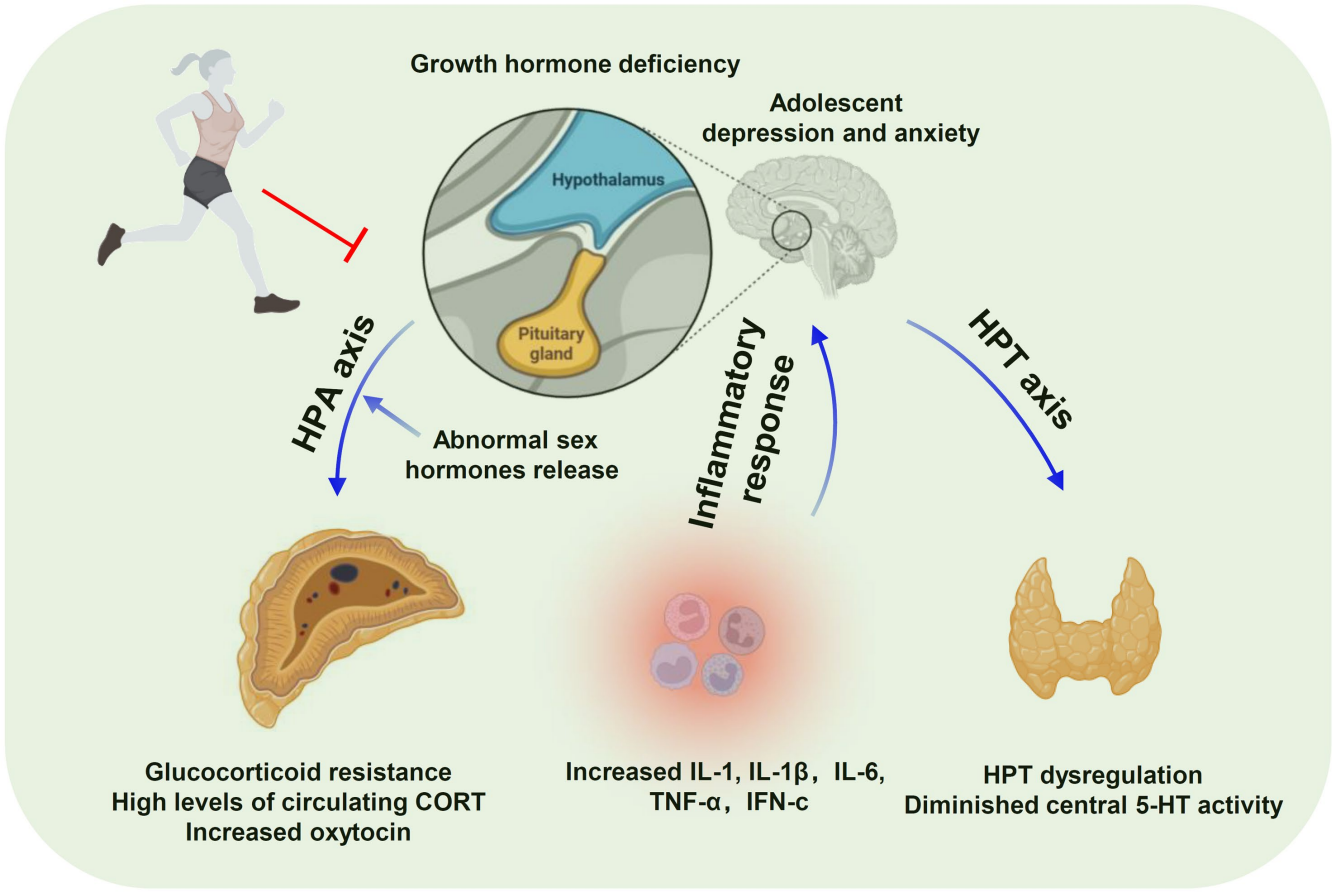
Exercise alleviates the pathological changes in anxiety and depression.

Among adolescents with depression and anxiety, there is substantial evidence that physical activity positively impacts adolescent mental health symptoms and that exercise has the potential as a diagnostic treatment for depression. Figure 1 summarizes the fundamental changes in anxiety and depression according to previous studies. The HPA axis is a hallmark factor in the neurophysiology of adolescent depression and anxiety (e.g., neurotrophic factors, neurotransmitters, hormones), and neuroimaging studies may be helpful. However, the number of relevant experimental studies is limited, and the studies on the anti-depressant effects of exercise have focused more on adults with exercise interventions in humans or mice, rats, and environment-related depression in adulthood. Little attention has been paid to depression and anxiety in the adolescent or child populations while existing studies are reviews or meta-analyses. Therefore, the efficacy of physical activity in young people (12–25 years) experiencing depression and anxiety, especially at the clinical level, remains to be determined. Moreover, the effects of physical exercise on depression and anxiety depend on several parameters of exercise training, including frequency, intensity, and types of exercise. Therefore, future research should prioritize the protocol design and incorporation of reasonable randomized controlled exercise intervention trials to support exercise against adolescent depression and anxiety. The lack of this information is also the a limitation of this review.

## Author contributions

XL and SH drafted the manuscript. SH and LY edited and revised the manuscript. All authors contributed to the article and approved the submitted version.

## Funding

This study was supported by the Hunan Province Higher Education Reform Project (HNJG-2020-1338 and HNJG-2022-0225).

## Conflict of interest

The authors declare that the research was conducted in the absence of any commercial or financial relationships that could be construed as a potential conflict of interest.

## Publisher’s note

All claims expressed in this article are solely those of the authors and do not necessarily represent those of their affiliated organizations, or those of the publisher, the editors and the reviewers. Any product that may be evaluated in this article, or claim that may be made by its manufacturer, is not guaranteed or endorsed by the publisher.

## Figures and Tables

**Table 1:** 

ACTH	Adrenocorticotropic hormone
AE	Aerobic exercise
BDNF	Brain-derived neurotrophic factor
CON	“No exercise” (control)
CRP	C-reactive protein
CVS	Chronic variable stress
CORT	Cortisol
CRF	Corticotropin-releasing factor
DHEA	Dehydroepiandrosterone sulfate
DST	Dexamethasone suppression test
GH	Growth hormone
GHD	Growth hormone deficiency
HIT	High-intensity interval training
HPA	Hypothalamic-pituitary-adrenal
HPT	Hypothalamic-pituitary-thyroid
IL	Interleukin
L-5-HTP	L-5-hydroxytryptamine
MCT	Moderate continuous training
MDD	Major depressive disorder
mPFC	Medial prefrontal cortex
p5-HT	Plasma serotonin
PTSD	Post-traumatic stress disorder
PVN	Paraventricular nucleus
REM	Rapid eye movement
SSRI	Selective serotonin reuptake inhibitors
T	Testosterone
TNF	Tumor necrosis factor
